# Predicting Mandibular Growth Potential Based on Cervical Vertebral Bone Age Using Lateral Cephalometric Radiographs in a Sample of the Saudi Population

**DOI:** 10.3390/diagnostics14192145

**Published:** 2024-09-26

**Authors:** Guna Shekhar Madiraju, Yousef Majed Almugla

**Affiliations:** 1 Faculty in Pediatric Dentistry, Department of Preventive Dental Sciences, College of Dentistry, King Faisal University, Al Ahsa 31982, Saudi Arabia; 2 Faculty in Orthodontics, Department of Preventive Dental Sciences, College of Dentistry, King Faisal University, Al Ahsa 31982, Saudi Arabia; yalmugla@kfu.edu.sa

**Keywords:** growth potential, mandible, cervical vertebral age, chronological age, cephalograms

## Abstract

Background: This study estimated the predictive accuracy of the mandibular growth potential based on cervical vertebral bone age using digital lateral cephalograms in a sample of the Saudi population. Methods: This study included digital lateral cephalograms of eighty subjects aged 10–21 years divided into adult and young groups. Cervical vertebral age was calculated by tracing and measuring the third and fourth cervical vertebrae on lateral cephalograms, and the mandibular growth potential was estimated using the Mito et al. regression equation. The accuracy of the calculated CVB age was verified via comparison with the chronological age. The data analyses included independent sample *t*-tests for testing the differences in mean values and Pearson correlation coefficients to examine the relationship between dependent and independent variables. Results: A significant difference was noted between the mean cervical vertebral age and chronological age in the young group for both males (*p* = 0.0003) and females (*p* = 0.033). The correlation coefficient between cervical vertebral age and chronological age in the young male group was higher (r = 0.934) than that in the young females (r = 0.254). Conclusions: The mandibular growth potential prediction based on CVB age using the regression equation of Mito et al. was applicable only to the young Saudi males. Further studies are needed to develop new multiple regression models to obtain cervical vertebral age more accurately for both genders in the Saudi population.

## 1. Introduction

An accurate assessment of the skeletal maturation age of an individual is essential in orthodontic diagnoses and treatment planning. Clinical decisions regarding the use of functional appliances or orthopaedic forces are based on growth considerations. Hence, an understanding of growth assessment and prediction is crucial in the practice of clinical orthodontics to coordinate orthopaedic–orthodontic procedures [1]. Amongst several biological indicators proposed to determine growth maturation, the assessment of skeletal age has been considered to be the best indicator with respect to the growth of facial bones [2]. Hand–wrist radiographs have been the most widely used method to predict pubertal growth spurt and estimate the proportion of growth remaining. However, the associated need for additional radiographs and radiation exposure prompted researchers to explore alternative assessment methods.

Whilst the cervical vertebral maturation (CVM) method is based on a judgement of vertebral morphology, diverse opinions exist in the literature regarding its effectiveness in assessing skeletal maturity [3,4,5,6,7]. Moreover, the reliability and reproducibility of the CVM assessment due to the difficulties in staging the cervical vertebrae morphology and classifying the third and fourth cervical vertebral bodies have been reported [8]. Bacetti et al. (2005) [1] had investigated the relation between the morphology of the second, third, and fourth cervical vertebrae (CV2–CV3–CV4) and the mandibular length in lateral cephalograms and improved the CVM method, originally given by Lemparski [9]. 

Cervical vertebral bone (CVB) age is determined objectively by evaluating skeletal maturation using routine diagnostic lateral cephalometric radiographs by measuring the maturational changes in cervical vertebrae [10,11,12]. Studies have reported CVB age to be an effective and equally reliable method as the hand–wrist radiograph method for assessing skeletal maturity and estimating the mandibular growth potential [5,6,7,12,13]. Mito et al. (2002) [11] and Caldas et al. (2007) [14] had suggested using methods depending on the equation for calculating the CVB age. The calculated CVB age and actual growth of the mandible were computed using a regression analysis to develop a formula for predicting the mandibular growth potential. CVB age was used to predict the mandibular growth potential in previous studies [13,15,16]. However, since racial and ethnic differences exist among various population groups, one equation might not be applicable for other population groups. 

Studies reporting on skeletal assessments using the CVB age and mandibular growth potential are very scarce in the Saudi population. The purpose of this study was to estimate the predictive accuracy of the mandibular growth potential, based on cervical vertebral bone age determined by using the regression equation given by Mito et al. (2002), in a sample of the Saudi population from the Eastern province region. The accuracy of the calculated CVB age was verified by comparing it with the chronological age.

## 2. Materials and Methods

This retrospective cross-sectional study included standard digital lateral cephalometric radiographs selected from the archives of patients who had attended the orthodontic and paediatric dental clinics in a university dental hospital. These radiographs were obtained for diagnosis and treatment planning. Only the age and gender of the subjects were recorded from the database. Lateral cephalograms of the patients obtained during the study period from March 2022 to July 2023 were selected, using the convenience sampling method, based on the following inclusion criteria: subjects aged 10–21 years with Saudi origin; requiring orthodontic treatment with a class I skeletal pattern; and radiographs presenting good quality and visualization of anatomical landmarks, specifically the third and fourth cervical vertebral bodies. Patients with any congenital tooth anomalies or systemic diseases that could affect normal craniofacial growth and development such as nutritional disturbance, endocrine disorders or syndromes, evidence of previous orthodontic treatment, anomalies in vertebral morphology, and radiographs of poor quality were excluded. The study protocol was reviewed and approved by the Institutional review board and was conducted in accordance with the ethical standards laid down in the 1964 Declaration of Helsinki. Written informed consent was obtained from the subjects for the use of radiographic data for research purposes. The chronological age was calculated from the birth date documented in the subject’s data record. All lateral cephalometric radiographs had been taken in accordance with international standards.

A total of 80 subjects met the inclusion criteria and were included in this study. The male and female groups were divided into two groups each, one group to calculate the predicted mandibular growth potential using CVB age (young group) and the other to compare the predicted values with the actual values (adult group). The young group (10–13 years) included subjects in the initial stage of the pubertal growth period, and the adult group (18–21 years) included those in the final stage of growth in the late adolescence or early adulthood stage.

**Figure 1:**
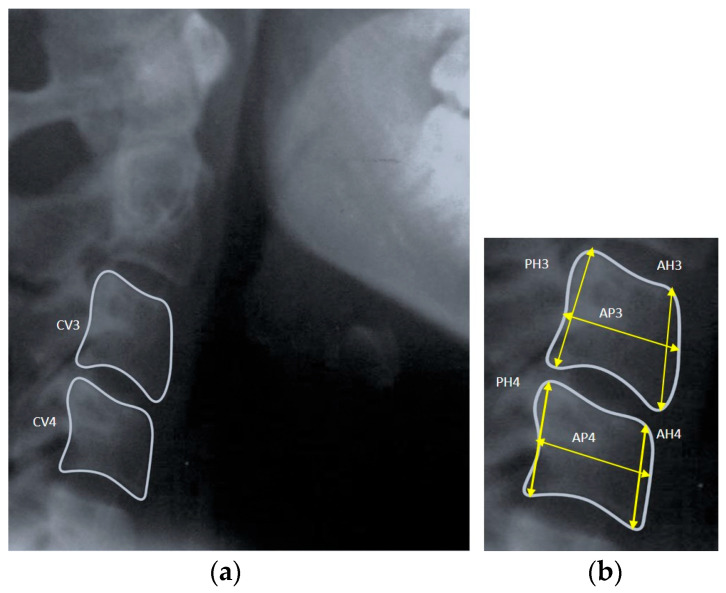
(**a**) Picture depicting measurements of the third and fourth cervical vertebrae on lateral cephalograms; (**b**) magnified view of CV3 and CV4.

A single well-experienced investigator blinded to the age and gender of the subjects obtained all the measurements on the digital lateral cephalograms. For each young subject, the anatomical landmarks were marked on the third and fourth cervical vertebral bodies (CV3 and CV4) on the lateral cephalograms, and geometric measurements were obtained for the anterior vertebral body height (AH), vertebral body height (H), posterior vertebral body height (PH), and anteroposterior vertebral body length (AP) [Figure 1a,b]. Then, the cervical vertebral bone age was calculated based on the following method described by Mito et al. (2002) [11]:

CVA (years) = –0.20 + 6.20 × AH3/AP3 + 5.90 × AH4/AP4 + 4.74 × AH4/PH4

where CVA is the cervical vertebral bone age, AH3 is the anterior vertebral body height of CV3, AP3 is the anteroposterior vertebral body length of CV3, AH4 is the anterior vertebral body height of CV4, AP4 is the anteroposterior vertebral body length of CV4, and PH4 is the posterior vertebral body height of CV4.

For each young subject, the mandibular growth potential was predicted from the cervical bone age by using the following equation [11]:

Mandibular growth potential (in mm) = −2.76 × cervical vertebral bone age + 38.68

**Figure 2:**
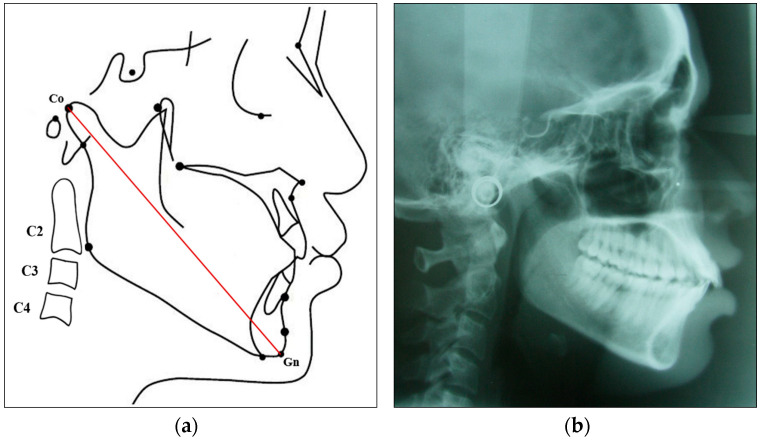
(**a**) Picture depicting cephalometric measurement from the condylion (Co) to the gnathion (Gn). (**b**) Lateral cephalogram of a subject with the skeletal class I pattern.

The linear distance from the condylion (Co) to the gnathion (Gn) was used to determine the total mandibular length (Co-Gn) for both genders in the adult and the young groups [Figure 2a,b]. The actual mandibular growth potential (in mm) of the adult group subjects was calculated by subtracting the mean mandibular length (Co-Gn) of the young group from the mean mandibular length (Co-Gn) of the adult group for both genders.

The mean value (mean ± SD) of the predicted mandibular growth potential for each gender in the young group was then compared with the mean value of the actual mandibular growth potential in the corresponding adult group. The intra-examiner reproducibility was tested by performing the measurements on ten randomly selected cephalograms from the study groups with an interval of two weeks. The intra-examiner correlation coefficient (ICC) score ranged from 0.89 to 0.91 (excellent).

### Statistical Analysis

The data were analysed using the SPSS software (Statistical Package for Social Sciences, version 20.0, IBM Corporation, New York, NY, USA). The data distribution was tested for normality. A comparison of the mean value of the actual growth potential in the adult group with the mean value of the predicted mandibular growth potential in the corresponding young group was performed to detect significant mean differences for both genders, using independent sample *t*-tests at a 95% confidence interval. Pearson correlation coefficients (r) were used to examine the relationship between dependent and independent variables. All statistical tests were two-sided, and a *p* value less than 0.05 was considered statistically significant.

## 3. Results

The final study sample consisted of digital lateral cephalograms of 80 subjects (40 males and 40 females) divided into two groups—young and adult groups—with equal distribution of gender. The age range of the young group was 10–13 years for both males (*n* = 20, average age 11.31 ± 0.56 years) and females (*n* = 20, average age 10.80 ± 0.35 years), while for the adult group, it was 18 to 21 years for both males (*n* = 20, average age 19.07 ± 0.55 years) and females (*n* = 20, average age 18.97 ± 0.45 years). 

The mean values (±SD) of chronological age in both the adult and young groups and the CVB age for the young group are presented in Table 1. A significant difference was noted between the mean CVB age calculated in the young group for both males (*p* = 0.0003) and females (*p* = 0.033) when compared to the mean chronological age of each respective young group, using independent sample *t*-tests.

The mean values of the mandibular length measured for both the adult and young groups and the calculated mandibular growth potential of the young group are shown in Table 2. The mean value of the actual mandibular growth potential of the adult group males and females was 9.16 ± 0.112 mm and 7.82 ± 0.06 mm, respectively. A comparison of the mean values of the actual mandibular growth potential of the adult group and predicted mandibular growth potential in the corresponding young group showed no statistically significant difference for both males (*p* = 0.801) and females (*p* = 0.279) (Table 3).

**Figure 3:**
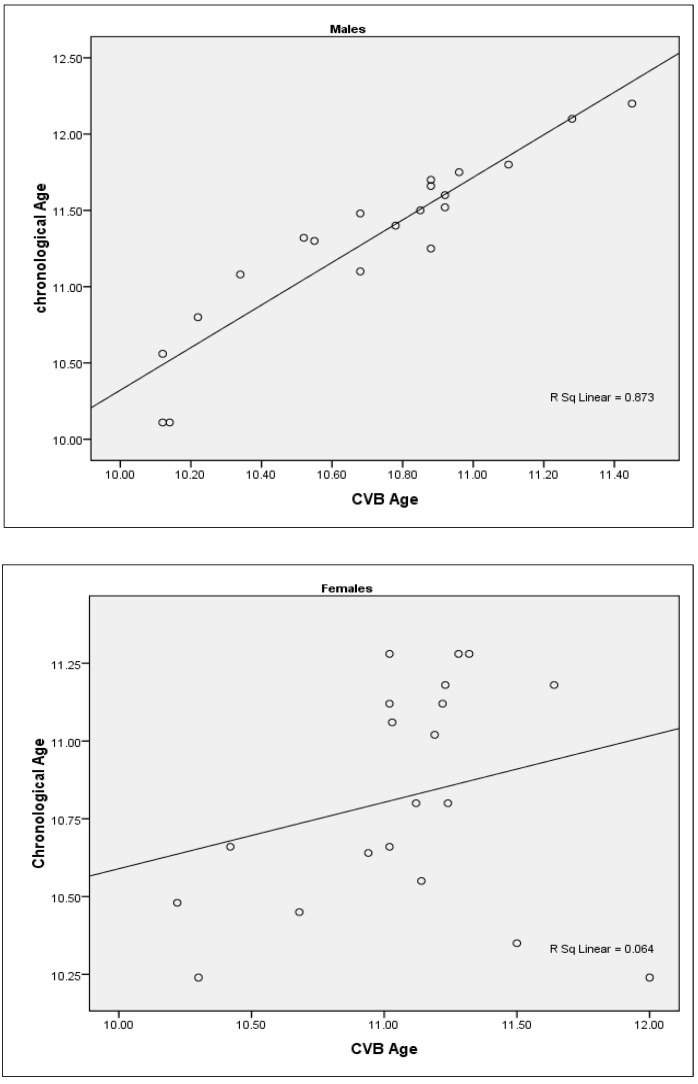
Scatter plots showing correlation between cervical vertebral bone age (CVB) and chronological age in the young male and female groups.

When the CVB age was correlated with the chronological age, the correlation coefficient between CVB age and chronological age in the young male group was higher and significant (r = 0.934; *p* = 0.000), while in the female group, a non-significant and weak correlation was found (r = 0.254; *p* = 0.280). This indicates that CV age closely approximates the chronological age in males better than in females (Table 4) [Figure 3].

## 4. Discussion

The prediction of mandibular growth potential has been found to be valuable in orthodontic treatment planning. The various methods used to predict mandibular growth potential using skeletal maturation as an indicator include the multiple regression method, the growth potential method, the ossification events method, and the growth chart and growth percentage methods. Regression equation analyses are the most widely used method for predicting mandibular growth potential. Mandibular growth potential has been predicted using CVB age as consistently and reliably as hand–wrist radiographs [13,17]. In the present study, CVB age and growth potential were calculated using the Mito et al. method, as it was suggested to be suitable for all classes of malocclusions [13]. This study objectively evaluated the predictive accuracy of the mandibular growth potential based on CVB age determined using the Mito et al. regression method in a sample of the young Saudi population from the AlAhsa region of Eastern Saudi Arabia.

Previous studies have reported a significant correlation between the hand–wrist and CVM methods and suggested that cervical vertebrae could offer an alternative method for assessing skeletal maturity without the need for additional hand–wrist radiographs [17,18]. Moshfeghi et al. (2013) [6] in a longitudinal study on Iranian girls aged 9–11 years had shown that CV dimensions could predict accurate mandibular growth potential. Chen et al. (2005) [19] reported an accurate estimation of the mandibular growth potential using CV measurements. Schoretsaniti et al. (2021) [20] in a recent study noted that the CVM method was unreliable in recognizing pubertal growth spurts and hence suggested that CVM should be used as an adjunct to other methods to obtain a more accurate estimation. Moreover, a lack of reproducibility among evaluators was reported as a major limitation of the CVM method. Another study in Saudi Arabia investigated a new CVM angular approach, involving the lower border concavity of CV bodies and reported that it was valid for the assessment of skeletal maturation in growing boys and suggested that this method could be used to estimate the pubertal growth peak [21].

Morphometric changes in the cervical vertebrae have been indicated at each stage of skeletal development from birth to maturation, whilst the period of maximal increment in mandibular growth has been demonstrated to occur between the CV3 and CV4 stages [22]. Previous research had used morphological changes in CV2, CV3, and CV4 to calculate skeletal maturation and suggested that the CV3 and CV4 bodies are adequate as CV2 revealed limited morphologic changes and morphometric difficulties [10]. Baidas (2012) [23] investigated the relation between the chronological age and CVM stages in Saudi adolescents and reported that the CVM method can be used as a maturity indicator of pubertal growth spurt better than chronological age. The present study analysed cephalometric radiographs to measure the CV3 and CV4 dimensions for calculating the CVB age and evaluate the mandibular growth potential in a sample of Saudi individuals. The CVB age calculated in the present study, according to Mito et al. method, was 10.71 ± 0.38 and 11.07 ± 0.42 years for the young male and female groups, respectively. The calculated CVB age was substituted in the regression equation to obtain the predicted mandibular growth potential in the young subjects. The CVB age was not calculated for the adult group as they would have completed their growth without any remaining mandibular growth potential.

Chronological age has been considered to be an inaccurate indicator to predict skeletal maturation [18]. Similarly, in this study, significant differences between the chronological age and CVB age were noted for both young male (*p* = 0.0003; t = 3.977) and female group subjects (*p* = 0.003; t = 3.148), using independent sample *t*-tests. On the contrary, other researchers have inferred that chronological age could be a useful predictor of mandibular growth potential or the onset of mandibular growth peak [13,24]. Studies conducted in the Brazilian and Saudi populations had reported no significant differences between CVB age and chronological age [12,15]. Chronological age was not used to calculate mandibular growth potential in the present study.

This study showed a strong correlation between CVB age and chronological age in young male group subjects (r = 0.93; *p* = 0.000), while in the young female group, a weak correlation was noted (r = 0.254; *p* = 0.280). Baidas (2012) [23] had used an improved version of the CVM method [1] and found a high correlation between chronological age and CV maturation in both genders. Chandrasekar et al. (2020) [25] found a moderate to strong positive correlation and no statistically significant difference between CVB age and chronological age in a sample of the south Indian population for both boys and girls. However, another research in an Indian population group demonstrated a statistically significant weak correlation of chronological age with CVB age and mandibular growth potential for both genders [16]. The differences in the findings seen in these studies could be due to variations in the sample size selection, race, and ethnicity of the population group under study. Our study sample for the young group was in the range of 10 to 12 years, whilst other studies had used a wider range of subjects’ ages, which could have affected the correlation and ability to detect skeletal maturity accurately. Moreover, gender-related differences in skeletal maturity have been reported in the literature [23,26].

Gender has been reported to play a role in influencing the timing of adolescent growth spurts and hence warrants consideration during clinical decision making regarding the timing of orthodontic treatment. The pubertal growth peak has been observed during the period ranging from 9 to 18 years of age [27]. Previous research in Saudi Arabia had reported the tendency for late skeletal maturity in Saudi subjects [23,28]. In the present study, the selected age ranges (9–21 years) were wide enough to ensure inclusion of growth peaks in the sample.

The accurate marking of landmarks is essential to assess maximal changes in the mandibular length. Previous studies had used different landmarks or reference points to determine the mandibular length, such as the pogonion, articlulare, condylion, and gnathion [6,19,22]. In the present study, the mandibular length was measured as the linear distance between the condylion and gnathion (Co-Gn), similar to that used by other researchers [1,15,22]. Moshfeghi et al. (2013) [6] in a study on Iranian girls had used the distance between the articulare and pogonion to measure the mandibular length and developed an equation which showed better prediction accuracy for mandibular growth potential compared to the method of Mito et al. (2002) [11]. Hence, an assessment of methodology used in various studies is essential for proper interpretation of the results. 

The regression equation developed by Mito et al. (2002) to obtain CVB age has been found be discrete in other population groups. The results were valid for Brazilian females but not males [14], whilst in the present study, the results were found valid for males but not females. Verma et al. (2021) [16] indicated that the Mito et al. method was valid for both genders in the south Indian population. These variations in the results between studies may be due to a variety of factors affecting the growth peak, including genetics, race, ethnicity, gender, environment, and lifestyle [29].

Variations in the amount and direction of pubertal mandibular growth across different races and gender have been reported [30]. Mandibular growth rotations are a reflection of differential growth in anterior and posterior face heights. Previous studies had revealed that y-axis and SN-MP angle measurements aid in the assessment of the direction of mandibular growth and mandibular rotation [31,32]. However, in the present study, the effect of mandibular rotation on the growth potential and mandibular length could not be assessed. Further studies should investigate the possible effect of rotation on the mandibular growth potential. 

An accurate estimation of MGP in the growth stage aids clinicians in predicting the amount of skeletal change attained, as functional appliance therapy produces greatest responses when the mandibular growth is at its peak during a pubertal growth spurt [2,16]. This is specifically useful in orthodontic clinical situations to initiate early orthodontic treatment for patients with class III malocclusion and in planning growth modification procedures for class II malocclusions and orthognathic surgery in severe skeletal deformity cases. Moreover, the orthodontic treatment plan varies for different skeletal classes, especially class III individuals who present comparatively longer durations of adolescent growth peaks, leading to increased mandibular growth [33]. The effect of skeletal class on mandibular growth potential could not be assessed in the present study. Another limitation in the present study includes the sample size derived from a university hospital setting, which may not represent the general population and hence limits the generalizability of the findings. Further research involving a larger sample is needed for more reliable conclusions. In addition, various skeletal patterns should be studied for analysing the proportion of incremental mandibular growth. Although longitudinal rather than cross-sectional studies might provide a better understanding of the results, ethical concerns regarding additional exposure to x-rays warrants consideration of this approach.

## 5. Conclusions

The cervical vertebral bone age method of predicting the mandibular growth potential was found applicable only to the young Saudi males. The predicted mandibular growth potential in the young group did not differ significantly from the actual mandibular growth potential in the corresponding adult group for both genders. Chronological age showed a statistically significant strong correlation with CVB age only in young Saudi males. Further research is imperative to develop new multiple regression models to obtain CVB age and predict the mandibular growth potential more reliably for both genders in the Saudi population. 

## Figures and Tables

**Table 1:** Descriptive statistics of chronological age and bone age in different groups.

Groups	Subgroup	Chronological Age (years)			CVB Age (years)		
		Mean ± SD	95% CI for Mean		Mean ± SD	95% CI for Mean	
			Lower Limit	Upper Limit		Lower Limit	Upper Limit
Adult Group	Males	19.07 ± 0.55	18.82	19.33	--	--	--
	Females	18.97 ± 0.45	18.76	19.18	--	--	--
Young Group	Males	11.31 ± 0.56	11.05	11.58	10.71 ± 0.380	10.535	10.891
	Females	10.81 ± 0.35	10.65	11.01	11.07 ± 0.42	10.875	11.277

CI: confidence interval; SD: standard deviation; CV: cervical vertebrae; independent sample *t*-test.

**Table 2:** Descriptive statistics of mandibular length and mandibular growth potential in different groups.

Groups	Subgroup	Mandibular Length (Co-Gn) (mm)			Mandibular Growth Potential (mm)		
		Mean ± SD (mm)	95% CI for Mean		Mean ± SD (mm)	95% CI for Mean	
			Lower Limit	Upper Limit		Lower Limit	Upper Limit
Adult Group	Males	113.99 ± 1.70	113.19	114.79	--	--	--
	Females	108.01 ± 1.56	1.073	1.087	--	--	--
Young Group	Males	104.83 ± 1.59	104.08	105.57	9.10 ± 1.05	8.61	9.59
	Females	100.19 ± 1.50	99.48	100.89	8.11 ± 1.18	7.55	8.66

CI: confidence interval; SD: standard deviation; CV: cervical vertebrae; independent sample *t*-test.

**Table 3:** Gender comparison of mandibular growth potential in adult and young groups.

Gender	Groups	Mandibular Growth Potential (mm) Mean ± SD	SEM	*p*-Value
Males (*n* = 40)	Adult group (actual)	9.16 ± 0.112	0.023	0.801
	Young group (calculated)	9.10 ± 1.054	0.235	
Females (*n* = 40)	Adult group (actual)	7.82 ± 0.060	0.013	0.279
	Young group (calculated)	8.11 ± 1.183	0.263	

*p* > 0.05: not significant. SD: standard deviation; SEM: standard error of mean.

**Table 4:** Correlation of cervical vertebral bone age (CVB) with chronological age (CA) in the young male and female groups.

Young Group (*n* = 40)		Pearson Correlation (r)	*p* Value
Males	CVB-CA	0.934	0.000
Females	CVB-CA	0.254	0.280 *

* *p* > 0.05: not significant.

## Data Availability

The data presented in this study are available from the corresponding author on request.
